# Transition From Parenteral to Enteral Nutrition and Postnatal Growth in Very Preterm Infants During Their First 28 Days of Life

**DOI:** 10.3389/fped.2022.775667

**Published:** 2022-03-10

**Authors:** Na Wang, Jia Zhang, Bo Wang, Zhangbin Yu, Shuping Han, Huaiyan Wang, Rongrong Chen, Li Gu, Yan Gao, Weiwei Hou, Xingxing Lu

**Affiliations:** ^1^Department of Pediatrics, The Affiliated Suqian First People's Hospital of Nanjing Medical University, Suqian, China; ^2^Department of Neonatology, Nanjing Maternity and Child Health Care Hospital, Women's Hospital of Nanjing Medical University, Nanjing, China; ^3^Department of Neonatology, Changzhou Maternity and Child Health Care Hospital, Changzhou, China; ^4^Department of Neonatology, Nantong Maternity and Child Health Care Hospital, Nantong, China; ^5^Department of Neonatology, Lianyungang Maternity and Child Health Care Hospital, Lianyungang, China; ^6^Department of Neonatology, Northern Jiangsu People's Hospital, Yangzhou, China

**Keywords:** enteral nutrition, parenteral nutrition, premature [MeSH], growth, first 28 days of life

## Abstract

**Background:**

Nutrition practices for preterm infants during the first few weeks of life can be divided into three phases: the parenteral nutrition (PN), enteral nutrition (EN), and transition (TN) phases; the TN phase includes both PN and EN. Our purpose was to analyze nutrition practices for very preterm infants during the TN phase and their association with the infants' growth during the first 28 days of life.

**Methods:**

Data from 268 very preterm infants <32 weeks old from six neonatal intensive care units were analyzed retrospectively. The TN phase was defined as enteral feedings of 30-120 ml/kg/d. Postnatal growth failure (PGF) was defined as a 28-day growth velocity <15 g/kg/d. Differences in protein and energy intake between the PGF and non-PGF groups during the TN phase were calculated, and risk factors for PGF were identified using multivariate regression analysis.

**Results:**

The total protein (parenteral + enteral) intake during the TN was 3.16 (2.89, 3.47) g/kg/d, which gradually decreased as the enteral feeding volume increased in the TN phase. The total energy (parenteral + enteral) intake during the TN phase was 115.72 (106.98, 122.60) kcal/kg/d. The PGF group had a lower total protein intake (parenteral + enteral) than the non-PGF group had [3.09 (2.85, 3.38) g/kg/d vs. 3.27 (3.06, 3.57) g/kg/d, *P* = 0.007, respectively]. No significant difference was found in energy intake during the TN phase. The variables associated with PGF included a lower total protein (parenteral + enteral) intake, a smaller day of age at the end of the TN phase, and a higher birth weight *z*-score.

**Conclusion:**

Increasing the total protein intake (parenteral + enteral) during the TN could reduce the incidence of PGF.

## Introduction

Postnatal growth failure (PGF) is prevalent among very preterm infants (1, 2), and the risk of developing adverse metabolic and neurodevelopmental outcomes later in life is high (3). In a longitudinal cohort study of the long-term growth of preterm infants, Andrews et al. (4) concluded that early PGF can be prevented. Hence, the importance of early nutritional management for the prevention of early growth failure among preterm infants has been gradually recognized (1, 5). The implementation of an enhanced parenteral nutrition (PN) protocol was found to promote growth, but it was also associated with an increased prevalence of severe hyperglycemia and higher mortality (6–9). Appropriate postnatal growth is more likely to be followed by better long-term outcomes (10, 11); therefore, it is particularly important for preterm infants to achieve appropriate nutrition intake and growth during their first few weeks of life.

Actual nutrition practices for preterm infants during their first few weeks of life can be divided into three phases: the parental nutrition (PN), enteral nutrition (EN), and transition (TN) phases, with the TN including both the PN and EN (12). Nutrition during the TN phase is a new challenge for neonatal physicians because it involves the transformation of parenteral amino acid (AA) to enteral protein (13–17). Liotto et al. (14) found that intensified nutritional management during the TN improved the postnatal growth of preterm infants. Moreover, a high incidence of poor growth during the TN predicted growth failure at discharge. However, poor growth during the PN phase was not significantly associated with growth failure at discharge (18).

Martin et al. (19) reported improved growth during the neonatal period by optimizing the early nutrition of preterm infants, but the relationship between nutrition during the TN phase and growth during the neonatal period remains unknown. The purpose of our study was to assess the nutritional status of preterm infants during the TN phase and examine its association with growth during the neonatal period.

## Materials and Methods

### Study Design and Population

This retrospective multicenter cohort study included six tertiary-care neonatal intensive care units (NICUs). Four NICUs were located in maternal and child health hospitals in Jiangsu, China: Nanjing Maternal and Child Health Hospital/Women's Hospital of Nanjing Medical University, Changzhou Maternal and Child Health Hospital, Nantong Maternal and Child Health Hospital, and Lianyungang Maternal and Child Health Hospital. Two NICUs were in general hospitals: Northern Jiangsu People's Hospital and the Affiliated Suqian First People's Hospital of Nanjing Medical University, both of them in Jiangsu, China.

Participants were recruited from January to December 2019. Infants included in the study were those born before 32 weeks of gestation, admitted to the NICU within 24 h of birth, and discharged after at least 28 days in the NICU. Infants who did not achieve full enteral feeding by the 28th day after birth were excluded.

### Nutrition Management

Infants received PN within 24 h after birth, and their initial glucose dose of 4-8 mg/kg/min was increased by 1-2 mg/kg/min, up to 11-14 mg/kg/min. Their AA intake was increased from 1.5-2.0 to 3.5-4.0 g/kg/d; lipids were started at 1.0 g/kg/d and increased by 0.5-1.0 g/kg/d, up to 3 g/kg/d.

Infants without contraindications to EN support should receive minimal enteral nutrition as early as possible after birth, with human milk or formula (10-20 ml/kg/d for 3-5 days). Full EN is achieved when the EN calorie intake is at least 80 kcal/kg/d. The target caloric value of full PN is 80-100 kcal/kg/d, and the target caloric value of full EN is 110-135 kcal/kg/d (20).

### Data Collection and Definitions

The TN phase began with the reduction of PN, i.e., an EN volume of 30-120 ml/kg/d (≥30 ml/kg/d, ≤ 120 ml/kg/d). The PN phase before the TN included a minimal volume of EN and a PN-only phase. Full EN was defined as an EN volume > 120 ml/kg/d or the absence of a PN infusion.

Data on the nutrient intake of all intravenous and enteral products during the TN phase and daily body weights were collected retrospectively to evaluate the intake of intestinal protein and energy, parenteral AA and energy, and total protein and energy. An EN volume of 30 ml/kg/d, which was considered equivalent to 30-39 ml/kg/d, was fed to the infants. When the feeding amount was the same for more than 1 day, the daily nutrient intake on these days was divided by the infant's body weight of that day, and the average of these nutrients in the feeding volume was calculated as the average nutrient composition of the feeding volume.

Growth indicators included birth weight, gestational age, time to regain birth weight, and growth velocity (GV). Being small for gestational age was defined as a birth weight less than the 10th percentile for gestational age (21). The birth weight *z*-score was calculated using an online preterm growth calculator (available at www.peditools.org.) (22), and the formula was: *z*-score = (the individual value – mean value)/standard deviation. The GV (from birth to the 28th day of life) was based on an exponential model of regaining birth weight. The formula was: GV = [1,000^*^ln (W_28_/W)]/(28-D); where W_28_ = weight on the 28th day of life, W = birth weight, and D = the day (of age) that birth weight was regained (23). The term PGF was defined as a 28-day GV <15 g/kg/d. The term non-PGF was defined as a 28-day GV ≥ 15 g/kg/d (24).

Feeding types included human milk and formula; human milk included mother's own milk (MOM) and donated human milk (DHM). When the volume of human milk reached 50-100 ml/kg/d, human milk fortifiers (HMFs) were added as supplements (20). The formula included: formula for preterm infants, extensively hydrolyzed infant formula and amino acid-based formula. The protein and energy components of the human milk were calculated in accordance with the respective nutrient reference values of transitional milk, mature milk, and donated milk (25, 26). The nutrient composition of the formula and HMFs were calculated according to the products' instructions (Table 1).

**Table 1:** Calculations of the protein and energy in the human milk/formula.

**Human milk/formula composition (per 100 ml)**		**Protein (g)**	**Energy (kcal)**
Human milk	Transitional milk (MOM)	1.50	67.00
	Half fortified transitional milk^a/b^	2.22/2.00	75.70/74.04
	Standard fortified transitional milk^a/b^	2.94/2.50	84.40/81.08
	Mature milk (MOM)	1.20	72.00
	Half fortified mature milk^a/b^	1.92/2.70	80.70/79.04
	Standard fortified mature milk^a/b^	2.64/2.20	89.40/86.08
	DHM	0.90	66.00
	Half fortified DHM^a/b^	1.62/1.40	74.70/73.04
	Standard fortified DHM^a/b^	2.34/1.90	83.40/80.08
Formula	Amino acid-based formula^c^	2.00	67.00
	Extensively hydrolyzed infant formula^d^	2.00	67.00
	Formula for preterm infants^e^	2.04	73.20

*MOM, mother's own milk; DHM, donated human milk*.

a/b *Human milk (maternal or donor) fortifiers, FM85®, Nestle, Swiss/Similac®, Abbott, US*;

c *Amino acid-based formula, Neocate®, Nutricia, Great Britain*,

d *Extensively hydrolyzed infant formula, Alfare®, Nestle, Netherlands*;

e *Formula for preterm infants, Prenan®, Nestle, Germany*.

Energy calculations of parenteral nutrition were as follows: glucose = 4 kcal/g (glucose injection); AA = 4 kcal/g (6% pediatric compound amino acid injection 18AA-I/19AA-I); and lipids = 9 kcal/g (20% medium and long-chain fat emulsion injection, C_8−24_Ve). During the PN-dominant TN phase, the amino acids in the PN were expressed as protein (1 g protein = 1.13 g AA) to calculate the total protein.

### Clinical Factors

The clinical factors associated with growth outcomes included bronchopulmonary dysplasia (BPD), with continuing oxygen requirements at 36 weeks corrected gestational age (27), necrotizing enterocolitis (NEC) ≥ grade 2 (28), intraventricular hemorrhage (IVH) ≥ grade 3/periventricular leukomalacia (29), confirmed sepsis (30) and invasive mechanical ventilation.

### Statistical Analysis

Continuous variables were expressed as median and interquartile range (IQR), and categorical variables were expressed as frequencies and percentages. The Mann-Whitney *U* and Chi-square tests or Fisher's exact test were used to analyze differences between groups. Variables with statistically significant differences in the univariate analysis were entered into the multivariate logistic regression model to determine the factors predicting PGF. *P* < 0.05 was considered statistically significant. All analyses were performed using SPSS statistical software, version 26 (IBM Corp. Armonk, NY).

## Results

### Study Population

In 2019, 742 preterm infants were treated in the six NICUs; 474 were excluded from the study: 294 because their length of stay was <28 days, 10 because complete data were not available, 2 because their admission to the NICU was >24 h after birth, and 168 because they had not completed the TN by the 28th day after birth. Thus, data from the remaining 268 infants were included in this study.

Participants' demographic characteristics and clinical outcomes are compared in Table 2. Birth weight and birth weight *z*-scores were significantly higher in the PGF group than those in the non-PGF group (*P* = 0.001*, P* = 0.013, respectively), but none of the clinical outcomes was significantly different.

**Table 2:** Comparisons of baseline characteristics and clinical outcomes.

	**Total (*n* = 268)**	**GV ≥15 g/kg/d (*n* = 71)**	**GV <15 g/kg/d (*n* = 197)**	***P-*value**
Birth weight *z*-score	0.42 (−0.08,0.89)	0.29(−0.39,0.70)	0.46(0.03,0.95)	0.013^a^^*^
Birth weight, median (IQR), g	1,290(1,160,1,490)	1,210(1,110,1,380)	1,330(1,170,1,525)	0.001^a^
Gestational age, median (IQR), weeks	29(28,30)	29(28,30)	29(28,30)	0.145^a^
Male sex, *n* (%)	133(49.6)	44(61.9)	89(45.1)	0.015^b^
Small for gestational age, *n* (%)	2(0.75)	1(1.41)	1(0.51)	0.460^b^
Time to regain birth weight, median (IQR), days	9(7,11)	8(7,11)	9(7.5,11)	0.518^a^
BPD, *n* (%)	87(32.46)	29(40.85)	58(29.4)	0.394^c^
NEC (≥grade 2), *n* (%)	18(6.72)	6(8.45)	12(6.09)	0.497^c^
IVH (≥grade 3)/PVL, *n* (%)	17(6.34)	3(4.23)	14(7.11)	0.394^c^
Confirmed sepsis, *n* (%)	20(7.46)	8(11.27)	12(6.09)	0.156^c^
Invasive mechanical ventilation, *n* (%)	59(22.01)	15(21.13)	44(22.34)	0.833^c^

*IQR, interquartile range; BPD, bronchopulmonary dysplasia; NEC, necrotizing enterocolitis; IVH, intraventricular hemorrhage; PVL, periventricular leukomalacia; GV, growth velocity; PGF, postnatal growth failure*.

a *Mann-Whitney U-test*;

b *Fisher's exact test*;

c *Chi-square test*.

* *P <0.05, significant difference between groups*.

### Comparisons of the Nutritional Data

Infants with PGF had a longer start time for the parenteral AA (*P* = 0.029), a smaller day of age at the end of the TN (*P* = 0.045), a lower AA intake during the PN phase (*P* = 0.01), and a lower total protein intake (PN + EN) during the TN phase (*P* = 0.007) than those without PGF. No significant difference was found in feeding types or other nutritional data during the TN phase (Table 3).

**Table 3:** Comparisons of the nutritional data and feeding types.

	**Total (*n* = 268)**	**GV ≥15g/kg/d (*n* = 71)**	**GV <15g/kg/d (*n* = 197)**	***P*-value^a^**
**Nutritional data, median (IQR)**				
Time to the initiation of parenteral AA, d	1(1,1)	1(1,1)	1(1,7)	0.029^*^
Initial dose of parenteral AA, g/kg/d	1.98(1.32,2)	1.98(1.43,2)	1.9(1.31,2)	0.109
Highest dose of parenteral AA, g/kg/d	3.51(3.11,3.84)	3.56(3.32,3.98)	3.5(3.06,3.79)	0.161
Time to the highest dose of parenteral AA, d	6(4,9)	6(4,10)	6(4,9)	0.963
Time to the cessation of parenteral AA, d	18(14,22)	19(14,24)	17(14,22)	0.129
Time to the initiation of parenteral lipids, d	2(1,2)	2(1,2)	2(1,2)	0.141
Initial dose of parenteral lipids, g/kg/d	1.03(0.97,1.49)	1.04(0.97,1.54)	1.03(0.96,1.43)	0.363
Highest dose of parenteral lipids, g/kg/d	3.38(2.96,3.67)	3.39(2.93,3.64)	3.33(2.96,3.70)	0.633
Time to the highest dose of parenteral lipids, d	6(4,10)	5(4,11)	6(4,9)	0.314
Time to the cessation of parenteral lipids, d	17(13,21)	18(13,24)	17(13,21)	0.271
Time to the initiation of enteral feeding, d	3(2,3)	3(2,3)	3(2,3)	0.108
Time to full enteral feeding of 120 ml/kg/d, d	15(12,20)	16(12,20)	15(12,9)	0.427
Time to full enteral feeding of 150 ml/kg/d, d	20(16,25)	20(17,25)	19(1626)	0.753
Time to supplementation with HMFs, d	17(13,21)	17(13,22)	17(3,21)	0.692
Feeding volume supplemented with HMFs, ml	121(107,143)	116(107,130)	124(106,146)	0.300
Time to the initiation of the TN, d	6(5,9)	8(5,10)	6(5,9)	0.103
Day of age at the end of the TN, d	17(14,22)	18(15,23)	17(13,20.5)	0.045^*^
Duration of the TN, d	10(8,14)	12(9,14)	10(8,14)	0.108
Highest energy intake during the PN phase, kcal/kg/d	75.10(91.30,102.25)	96.40(82.10,103.90)	88.90(74.07,101.38)	0.066
Highest AA intake during the PN phase, g/kg/d	3.6(3.1,3.9)	3.7(3.45,4.0)	3.6(3.07,3.8.0)	0.010^*^
Duration of the PN phase, d	5(4,8)	7(4,9)	5(4,8)	0.103
Total protein intake (PN + EN) during the TN, g/kg/d	3.16(2.89,3.47)	3.27(3.06,3.57)	3.09(2.85,3.38)	0.007^*^
Total energy intake (PN + EN) during the TN, kcal/kg/d	115.72(106.98,122.60)	118.39(109.29,122.54)	115.12(106.18,122.95)	0.371
**Feeding types**, ***n*** **(%)**				
Breast milk	146(54.4%)	40(56.3%)	106(53.8%)	0.714
Formula	13(4.8%)	4(5.6%)	9(4.5%)	0.750
Breast milk and formula	109(40.6%)	27(38%)	82(41.6%)	0.597

Total protein = parenteral amino acid *1.13 + enteral protein.

*d, days; GV, growth velocity; HMF, human milk fortification; PN, parenteral nutrition; EN, enteral nutrition; TN, transition phase; AA, amino acid; PGF, postnatal growth failure*.

a *P-values are for the Mann-Whitney U-test for continuous variables and the Chi-square or Fisher's exact test for categorical variables*.

* *P <0.05, significant difference between groups*.

**Figure 1:**
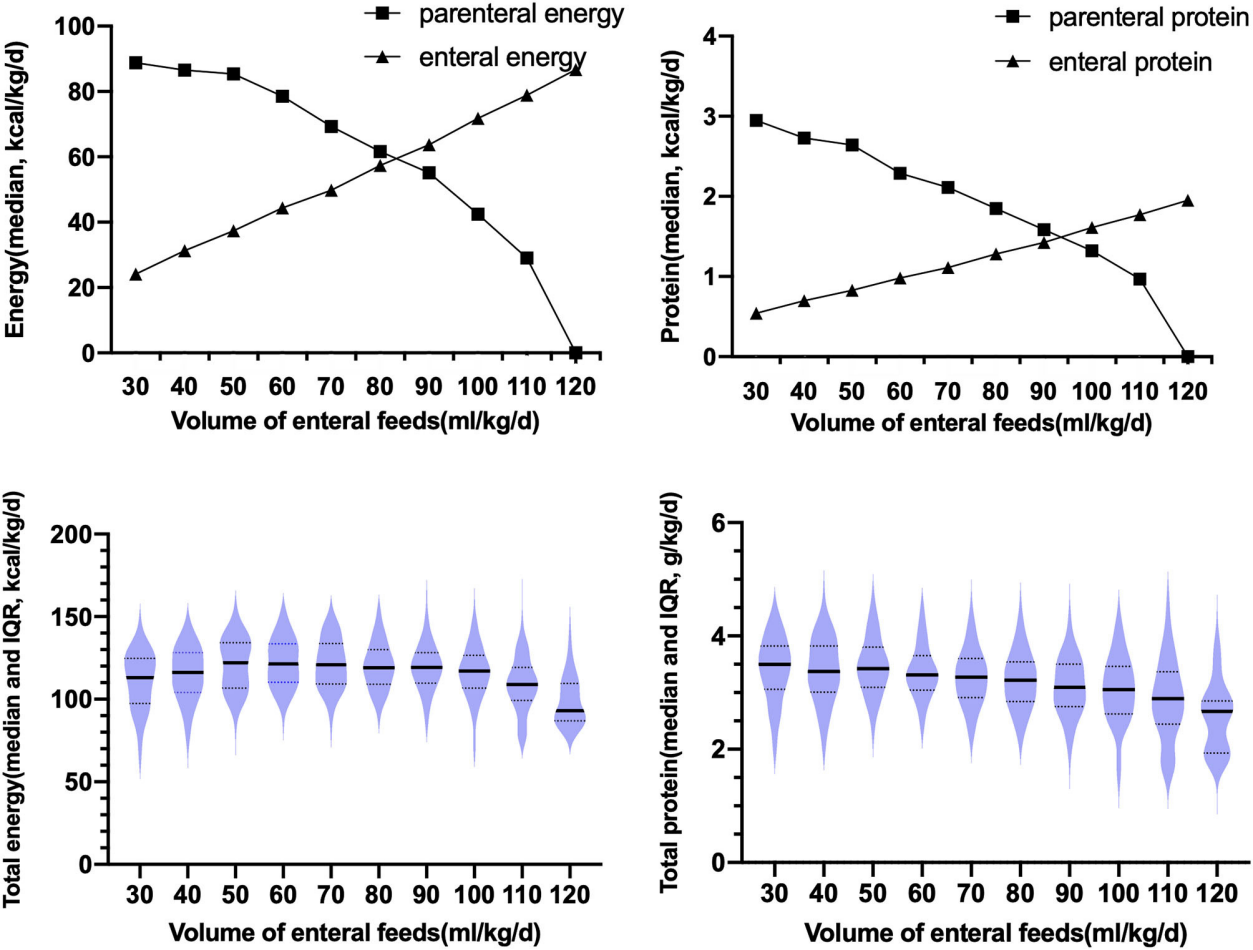
Changes in the parenteral and enteral protein intake, and the medians and interquartile ranges of the total parenteral and total enteral energy and protein intake during the TN phase; IQR, interquartile range.

During the TN phase, with the increase in the enteral feeding volume, the energy and protein provided by the PN gradually decreased, and the energy and protein provided by the EN gradually increased, as shown in Figure 1. When the enteral feeding volume reached 80 ml/kg/d, the protein and energy provided by the PN was equivalent to that provided by the EN. Total protein (parenteral + enteral) during the TN phase was 3.16 (2.89, 3.47) g/kg/d, which gradually decreased with the increase in the enteral feeding volume. The total energy (parenteral + enteral) intake during the TN phase was 115.72 (106.98, 122.60) kcal/kg/d.

Infants in the PGF group had a lower parenteral AA intake throughout the TN phase than those in the non-PGF group. Before the enteral feeding volume reached 80 ml/kg/d, the infants in the PGF group had a lower parenteral AA intake [2.85 (2.44, 3.20) g/kg/d vs. 3.07 (2.60, 3.46) g/kg/d, *P* = 0.025, respectively] and a lower enteral protein intake [0.82 (0.77, 0.89) g/kg/d vs. 0.84 (0.81, 0.92) g/kg/d, *P* = 0.03, respectively). After the enteral feeding volume reached 80 ml/kg/d, infants in the PGF group had a lower parenteral AA intake [1.30 (1.01, 1.69) g/kg/d vs.1.40 (1.20, 1.68) g/kg/d, *P* = 0.048, respectively]. No significant difference was found in the parenteral or enteral energy intake before or after the enteral feeding volume reached 80 ml/kg/d (*P* > 0.05).

### Predictors of Postnatal Growth Failure in the First 28 Days of Life

We performed a multivariate logistic regression analysis of the nutritional predictors of PGF during the first 28 days of life (Table 4). Three variables were kept in the model (*P* < 0.001): the total protein intake (PN + EN) during the TN (*P* = 0.005), the day of age at the end of the TN (*P* = 0.006), and the birth weight *z*-score (*P* = 0.017). For every 0.1 g/kg/d increase in the total protein intake during the early TN phase, the risk for 28-day growth failure decreased by 8.3%.

**Table 4:** Risk factors identified as predictors of PGF in very preterm infants in the multivariate analysis.

**Predictors of PGF**	**Adjusted OR**	**95%CI**	***P*-value**
Birth weight *z*-score	1.635	1.09-2.45	0.017^*^
Time to initiation of parenteral AA	3.634	0.99-13.33	0.052
Highest AA intake during the PN phase	1.42	0.69-2.91	0.34
Day of age at the end of the TN	0.905	0.84-0.97	0.006^*^
BPD	0.835	0.42-1.66	0.608
NEC ≥ grade 2	1.076	0.35-3.23	0.896
Total protein (PN + EN) during the TN	0.172	0.05-0.59	0.005^*^
Total energy (PN + EN) during the TN	1.018	0.97-1.06	0.428

*OR, odds ratio; CI, confidence interval; BPD, bronchopulmonary dysplasia; NEC, necrotizing enterocolitis; PN, parenteral nutrition; EN, enteral nutrition; TN, transition phase; AA, amino acid; PGF, postnatal growth failure*.

* *P <0.05, significant difference between groups*.

## Discussion

This study analyzed the nutrition data of very preterm infants from six NICUs, excluding data from preterm infants who were still in the TN phase at their 28th day of life. Analysis of the relationship between nutrition intake during the TN phase and the growth of very preterm infants during the first 4 weeks of life showed that lack of protein during the TN phase was associated with growth failure during the first 28 days. This finding differs from those of previous studies on the nutrition and growth of preterm infants (1, 10, 19, 26). Previous studies have reported that achieving adequate nutritional intake during the first few weeks of life is challenging for preterm infants, and a potential strategy to solve this problem is to start parenteral nutrition early, i.e., shortly after birth (31, 32). Our study found that preterm infants without PGF started parenteral nutrition earlier than infants with PGF did, and their amino acid intake during the PN phase was higher; however, neither factor predicted growth failure during the neonatal period. In our study, the nutrition of preterm infants was divided by nutritional phase rather than by postnatal period. We found that protein deficiency was most likely to occur in the TN phase. Therefore, total protein intake (PN + EN) during the TN can predict growth failure during the neonatal period, while total energy intake has no obvious correlation with growth during the neonatal period.

The median duration of the TN phase was 10 days, which is consistent with data from previous studies (33). The older the day of age at the end of the TN phase, the lower the risk of 28-day growth failure, which may be related to lack of enteral protein intake during the TN phase, resulting in a longer period of PN intake. Furthermore, our study found that the birth weight z-score was negatively correlated with the GV because the preterm infants with a lower birth weight z-score often had a higher parenteral amino acid intake during the PN-only and TN phases. This finding is consistent with the negative correlation between birth weight z-score and changes in the birth weight z-scores reported by Izquierdo et al. (2).

The introduction and promotion of the enteral feeding of preterm infants with NEC is a major modifiable risk factor (34), which undoubtedly affects the outcomes of preterm infants with NEC (35). Despite newer and gentler modes of ventilation, BPD remains a major cause of neonatal mortality and morbidity (36). Preterm infants with BPD often show better postnatal growth because of continued PN (37). Therefore, NEC, BPD, and the other clinical factors analyzed in this study may be related to the growth of preterm infants. Our study population included very preterm infants who had completed the TN phase during the neonatal period and achieved full enteral feeding. The inclusion criteria precluded neonates with severe clinical outcomes during the neonatal period; therefore, no adverse outcomes (e.g., NEC or BPD) were identified as risk factors in the multivariate analysis of 28-day growth failure.

In a systematic review of GV calculations, the most commonly used measurement for calculating growth was g/kg/d (38). In our study, we found that birth weight was an important variable affecting PGF. However, the GV was based on an exponential calculation combined with the time to regain birth weight (39). The formula itself contained two important variables: birth weight and the time to regain birth weight, which were beneficial for our growth evaluations (40, 41). Therefore, it is more accurate and reasonable to conclude that insufficient protein intake during the TN phase can predict growth failure during the neonatal period.

In our study, the TN phase was defined as 30 ml/kg/d ≤ the enteral feeding volume ≤ 120 ml/kg/d, and could be divided into early and late stages by an enteral feeding volume of 80 ml/kg/d. The definition of the TN phase remained controversial (13–18, 33). The starting point of the TN can be defined as an enteral feeding volume of 30 ml/kg/d because the progressive reduction of PN always occurred when the enteral feeding volume reached 30 ml/kg/d (after a non-nutritive feeding) (20). When the enteral feeding volume reached 120 ml/kg/d, the median total energy was 115.72 kcal/kg/d, which met the energy intake recommended by the Chinese Society of Parenteral and Enteral Nutrition (CSPEN) Guideline (20); thus, the end-point definition was also reasonable. Our study found that the period with the EN volume of 30-70 ml/kg/d was the PN-dominant TN, and the period with the EN volume of 80-120 ml/kg/d was the EN-dominant TN. At the point of 80 ml/kg/d (EN volume), the parenteral and enteral energy were both about 60 kcal/kg/d. Based on the point, the TN phase could be divided into early and late stages, which was consistent with the study of Brennan et al. (13).

The phase before the volume of enteral feeding that reached 30 ml/kg/d was the PN phase. In our study, the median of the amino acid intake during the PN phase was above 3.5 g/kg/d, which met the CSPEN Guideline's recommendation (20); however, the intake of the non-PGF group was significantly higher than that of the PGF group during this phase. Hence, a higher amino acid infusion may be beneficial to the growth of the newborn during the PN phase.

During the following TN phase, the median total protein intake gradually decreased; it was <3.5 g/kg/d. Moreover, the PGF group had a lower parenteral AA intake throughout the TN than the non-PGF group. Thus, the common phenomenon of insufficient protein intake during the TN phase predicted the 28-day growth failure, which further explains the association of insufficient protein intake during the TN with poor growth during the neonatal period. Our study found the cut-off point between the early and late stages of the TN phase (i.e., when the amount of enteral feeding was 80 ml/kg/d and the protein intake during the PN phase was greater than that of the EN phase, and after the volume of enteral feeding was 90 ml/kg/d and the protein intake of the EN phase gradually exceeded that of the PN phase) reflected the current situation of insufficient protein intake throughout the TN for very preterm infants. Therefore, during the TN phase, the total protein intake should reach 3.5 g/kg/d to promote better growth among preterm infants. Although human milk feeding has a lower protein content than formula feeding, human milk can promote the deposition of fat-free substances, resulting in better neurodevelopment. Therefore, human milk is still the preferred choice for preterm infants during the late TN phase (42). In order to increase the total protein intake while considering the individual differences of preterm infants, human milk fortifiers can be used as supplements, as appropriate, when the human milk volume reaches 50-100 ml/kg/d (14). At the same time, regulating the composition of the standardized PN solutions during the TN phase, including carbohydrates, fats, and amino acids, can achieve the goal of optimal nutrient intake to improve the growth of preterm infants during the neonatal period (13, 14). Moreover, considering the high mortality rate caused by hyperglycemia in extremely low-birth-weight infants, the optimal composition of the early PN phase to avoid PGF must be balanced against the risk for hyperglycemia (9).

One of the strengths of our study is its multicenter cohort with a large sample size. In addition, few studies have examined the relationship between TN nutrition, and early growth of preterm infants. We also reported the current situation of insufficient protein intake during the TN phase. However, there are some limitations. First, the growth assessment was based only on the infant's weight and the time to regain birth weight, without considering the infant's length or head circumference. Second, the study's retrospective observational design and lack of randomization limits the interpretation of the results. Furthermore, standardized nutrition guidelines for the TN phase had not been established before the study. Thus, future studies should examine the associations between the infants' nutrition during the TN phase (including carbohydrates, fats, and amino acids) and their long-term prognoses.

## Conclusion

The TN from PN to EN is a critical period for maintaining adequate growth among preterm infants. Our study showed that protein intake during the TN phase could predict growth during the neonatal period, suggesting that neonatal physicians should pay more attention to infants' total protein intake (PN + EN) during the TN phase, especially parenteral amino acid intake.

## Data Availability Statement

The original contributions presented in the study are included in the article/supplementary material, further inquiries can be directed to the corresponding author/s.

## Ethics Statement

This study was approved by the Research Ethics Committee of the Women's Hospital of Nanjing Medical University (Ethics Number: Ning Fulun [2016] No. 73). Written informed consent from the participants' legal guardians was not required for participation in this study, in accordance with national legislation and the institutions' requirements.

## Author Contributions

NW, ZY, and JZ contributed to conception and design of the study. NW and SH contributed to the acquisition and analysis of the data. BW, RC, LG, XL, HW, YG, and WH performed the statistical analysis. NW wrote the first draft of the manuscript. All authors contributed to manuscript revision, read, and approved the submitted version.

## Funding

This research was funded by the Nanjing Medical Science and Technology Development Foundation (ZKX19045) and Project supported by special disease cohort of Nanjing Medical University (NMUC2020037).

## Conflict of Interest

The authors declare that the research was conducted in the absence of any commercial or financial relationships that could be construed as a potential conflict of interest.

## Publisher's Note

All claims expressed in this article are solely those of the authors and do not necessarily represent those of their affiliated organizations, or those of the publisher, the editors and the reviewers. Any product that may be evaluated in this article, or claim that may be made by its manufacturer, is not guaranteed or endorsed by the publisher.

## References

[1] McKenzie BL Edmonds L Thomson R Haszard JJ Houghton LA. Nutrition practices and predictors of postnatal growth in preterm infants during hospitalization: a longitudinal study. J Pediatr Gastroenterol Nutr. (2018) 66:312–7. 10.1097/MPG.0000000000001747 28953525

[2] Izquierdo Renau M Aldecoa-Bilbao V Balcells Esponera C Del Rey Hurtado de Mendoza B Iriondo Sanz M Iglesias-Platas I. Applying methods for postnatal growth assessment in the clinical setting: evaluation in a longitudinal cohort of very preterm infants. Nutrients. (2019) 11:E2772. 10.3390/nu11112772 31739632 PMC6893690

[3] Luu TM Rehman Mian MO Nuyt AM. Long-term impact of preterm birth: neurodevelopmental and physical health outcomes. Clin Perinatol. (2017) 44:305–14. 10.1016/j.clp.2017.01.003 28477662

[4] Andrews ET Ashton JJ Pearson F Beattie RM Johnson MJ. Early postnatal growth failure in preterm infants is not inevitable. Arch Dis Child Fetal Neonatal Ed. (2019) 104:F235–41. 10.1136/archdischild-2018-315082 30135111

[5] Maas C Franz AR von Krogh S Arand J Poets CF. Growth and morbidity of extremely preterm infants after early full enteral nutrition. Arch Dis Child Fetal Neonatal Ed. (2018) 103:F79–81. 10.1136/archdischild-2017-312917 28733478

[6] Thoene M Anderson-Berry A. Early enteral feeding in preterm infants: a narrative review of the nutritional, metabolic, and developmental benefits. Nutrients. (2021) 13:2289. 10.3390/nu13072289 34371799 PMC8308411

[7] Törer B Hanta D Özdemir Z Çetinkaya B Gülcan H. An aggressive parenteral nutrition protocol improves growth in preterm infants. Turk J Pediatr. (2015) 57:236–41. 26701941

[8] Roelants JA Joosten K Van D Hulst JM Reiss I Vermeulen MJ. First week weight dip and reaching growth targets in early life in preterm infants. Clin Nutr. (2018) 37:1526–33. 10.1016/j.clnu.2017.08.023 28912010

[9] Stensvold HJ Strommen K Lang AM Abrahamsen TG Steen EK Pripp AH. Early enhanced parenteral nutrition, hyperglycemia, and death among extremely low-birth-weight infants. JAMA Pediatr. (2015) 169:1003–10. 10.1001/jamapediatrics.2015.1667 26348113

[10] Stephens BE Walden RV Gargus RA Tucker R McKinley L Mance M. First-week protein and energy intakes are associated with 18-month developmental outcomes in extremely low birth weight infants. Pediatrics. (2009) 123:1337–43. 10.1542/peds.2008-0211 19403500

[11] Sammallahti S Kajantie E Matinolli HM Pyhälä R Lahti J Heinonen K. Nutrition after preterm birth and adult neurocognitive outcomes. PLoS ONE. (2017) 12:e0185632. 10.1371/journal.pone.0185632 28957424 PMC5619810

[12] Roggero P Giannì ML Orsi A Amato O Piemontese P Liotto N. Implementation of nutritional strategies decreases postnatal growth restriction in preterm infants. PLoS ONE. (2012) 7:e51166. 10.1371/journal.pone.0051166 23227249 PMC3515560

[13] Brennan AM Kiely ME Fenton S Murphy BP. Standardized parenteral nutrition for the transition phase in preterm infants: a bag that fits. Nutrients. (2018) 10:170. 10.3390/nu10020170 29393903 PMC5852746

[14] Liotto N Amato O Piemontese P Menis C Orsi A Corti MG. Protein intakes during weaning from parenteral nutrition drive growth gain and body composition in very low birth weight preterm infants. Nutrients. (2020) 12:1298. 10.3390/nu12051298 32370158 PMC7282247

[15] Wang L Liu D Shen H Wang Y Han L He Z. Analysis of amino acid patterns with nutrition regimens in preterm infants with extrauterine growth retardation. Front Pediatr. (2020) 8:184. 10.3389/fped.2020.00184 32426308 PMC7212428

[16] Falciglia GH Murthy K Holl JL Palac HL Oumarbaeva Y Woods DM. Energy and protein intake during the transition from parenteral to enteral nutrition in infants of very low birth weight methods. J Pediatr. (2018) 202:38–43.e31. 10.1016/j.jpeds.2018.07.010 30195557

[17] Brennan AM Fenton S Murphy BP Kiely ME. Transition phase nutrition recommendations: a missing link in the nutrition management of preterm infants. JPEN J Parenter Enteral Nutr. (2018) 42:343–51. 10.1177/0148607116686289 28555514

[18] Miller M Vaidya R Rastogi D Bhutada A Rastogi S. From parenteral to enteral nutrition: a nutrition-based approach for evaluating postnatal growth failure in preterm infants. JPEN J Parenter Enteral Nutr. (2014) 38:489–97. 10.1177/0148607113487926 23674574

[19] Martin CR Brown YF Ehrenkranz RA O'Shea TM Allred EN Belfort MB. Nutritional practices and growth velocity in the first month of life in extremely premature infants. Pediatrics. (2009) 124:649–57. 10.1542/peds.2008-3258 19651583 PMC2859427

[20] Working Working Group of Pediatrics Chinese Society of Parenteral and Enteral Nutrition Working Working Group of Neonatology Chinese Society of Pediatrics Working Working Group of Neonatal Surgery Chinese Society of Pediatric Surgery. CSPEN guidelines for nutrition support in neonates. Asia Pac J Clin Nutr. (2013) 22:655–63. 10.6133/apjcn.2013.22.4.21 24231027

[21] Battaglia FC Lubchenco LO. A practical classification of newborn infants by weight and gestational age. J Pediatr. (1967) 71:159–63. 10.1016/S0022-3476(67)80066-0 6029463

[22] Chou JH Roumiantsev S Singh R. Peditools electronic growth chart calculators: applications in clinical care, research, and quality improvement. J Med Internet Res. (2020) 22:e16204. 10.2196/16204 32012066 PMC7058170

[23] Patel AL Engstrom JL Meier PP Kimura RE. Accuracy of methods for calculating postnatal growth velocity for extremely low birth weight infants. Pediatrics. (2005) 116:1466–73. 10.1542/peds.2004-1699 16322172

[24] Fenton TR Anderson D Groh-Wargo S Hoyos A Ehrenkranz RA Senterre T. An attempt to standardize the calculation of growth velocity of preterm infants—evaluation of practical bedside methods. J Pediatr. (2018) 196:77–83. 10.1016/j.jpeds.2017.10.005 29246464

[25] Cormack BE Embleton ND van Goudoever JB Hay WW Jr Bloomfield FH. Comparing apples with apples: it is time for standardized reporting of neonatal nutrition and growth studies. Pediatr Res. (2016) 79:810–20. 10.1038/pr.2016.26 26866908

[26] Cormack BE Jiang Y Harding JE Crowther CA Bloomfield FH. Relationships between neonatal nutrition and growth to 36 weeks' corrected age in elbw babies–secondary cohort analysis from the provide trial. Nutrients. (2020) 12:760. 10.3390/nu12030760 32183057 PMC7146349

[27] Jobe AH Bancalari E. Bronchopulmonary dysplasia. Am J Respir Crit Care Med. (2001) 163:1723–9. 10.1164/ajrccm.163.7.2011060 11401896

[28] Rich BS Dolgin SE. Necrotizing enterocolitis. Pediatr Rev. (2017) 38:552–9. 10.1542/pir.2017-0002 29196510

[29] Mukerji A Shah V Shah PS. Periventricular/intraventricular hemorrhage and neurodevelopmental outcomes: a meta-analysis. Pediatrics. (2015) 136:1132–43. 10.1542/peds.2015-0944 26598455

[30] Shane AL Sánchez PJ Stoll BJ. Neonatal sepsis. Lancet. (2017) 390:1770–80. 10.1016/S0140-6736(17)31002-4 28434651

[31] Kresch M Mehra K Jack R Greecher C. Sustaining improved nutritional support for very low birthweight infants. BMJ Open Qual. (2020) 9:e000672. 10.1136/bmjoq-2019-000672 32188738 PMC7078686

[32] Baillat M Pauly V Dagau G Berbis J Boubred F Fayol L. Association of first-week nutrient intake and extrauterine growth restriction in moderately preterm infants: a regional population-based study. Nutrients. (2021) 13:227. 10.3390/nu13010227 33466801 PMC7830065

[33] Alur P Kalikkot Thekkeveedu R Meeks M Hart KC Desai J Johnson M. Calorie intake is associated with weight gain during transition phase of nutrition in female extremely low birth weight infants. Biol Sex Differ. (2020) 11:16. 10.1186/s13293-020-00295-7 32293535 PMC7160909

[34] Christian VJ Polzin E Welak S. Nutrition management of necrotizing enterocolitis. Nutr Clin Pract. (2018) 33:476–82. 10.1002/ncp.10115 29940075

[35] Raturi S Zheng Q Daniel LM Shi L Rajadurai VS Agarwal PK. Nutritional intake and growth velocity in preterm extremely low-birthweight infants in Asia: are we doing enough? J Paediatr Child Health. (2017) 53:1199–207. 10.1111/jpc.13630 28833725

[36] Sahni M Bhandari V. Recent advances in understanding and management of bronchopulmonary dysplasia. F1000Res. (2020) 9:F1000. 10.12688/f1000research.25338.1 32704351 PMC7361502

[37] Dassios T Williams EE Hickey A Bunce C Greenough A. Bronchopulmonary dysplasia and postnatal growth following extremely preterm birth. Arch Dis Child Fetal Neonatal Ed. (2021) 106:386–91. 10.1136/archdischild-2020-320816 33334820

[38] Fenton TR Chan HT Madhu A Griffin IJ Hoyos A Ziegler EE. Preterm infant growth velocity calculations: a systematic review. Pediatrics. (2017) 139:e20162045. 10.1542/peds.2016-2045 28246339

[39] Fenton TR Griffin IJ Hoyos A Groh-Wargo S Anderson D Ehrenkranz RA. Accuracy of preterm infant weight gain velocity calculations vary depending on method used and infant age at time of measurement. Pediatr Res. (2019) 85:650–4. 10.1038/s41390-019-0313-z 30705399

[40] Goldberg DL Becker PJ Brigham K Carlson S Fleck L Gollins L. Identifying malnutrition in preterm and neonatal populations: recommended indicators. J Acad Nutr Diet. (2018) 118:1571–82. 10.1016/j.jand.2017.10.006 29398569

[41] Xu H Yang C Xu P. Observation on the efficacy and complications of intravenous nutrition strategy in premature infants with birth weight <1 500 g. Zhonghua Wei Zhong Bing Ji Jiu Yi Xue. (2019) 31:1395–400. 10.3760/cma.j.issn.2095-4352.2019.11.016 31898572

[42] Cerasani J Ceroni F De Cosmi V Mazzocchi A Morniroli D Roggero P. Human milk feeding and preterm infants' growth and body composition: a literature review. Nutrients. (2020) 12:1155. 10.3390/nu12041155 32326178 PMC7230190

